# First‐Line Immune‐Combination Therapy for Driver Gene‐Negative NSCLC With Brain Metastases: Real‐World Outcomes

**DOI:** 10.1111/1759-7714.70095

**Published:** 2025-05-28

**Authors:** Mengxing You, Lige Wu, Jiayu Liu, Hanqi Yuan, Zihe Wang, Xuezhi Hao, Puyuan Xing, Junling Li

**Affiliations:** ^1^ Department of Medical Oncology National Cancer Center/National Clinical Research Center for Cancer/Cancer Hospital, Chinese Academy of Medical Sciences and Peking Union Medical College Beijing China

**Keywords:** brain metastases, driver gene, immunotherapy, non‐small cell lung cancer, real‐world study

## Abstract

**Background:**

Optimal treatment for driver gene‐negative non‐small cell lung cancer (NSCLC) with brain metastases (BM) remains unclear, particularly regarding immune checkpoint inhibitor (ICI)‐based combinations and local BM therapy. Predictive biomarkers for intracranial efficacy are also undefined.

**Methods:**

This retrospective study analyzed driver gene‐negative NSCLC patients with BM treated with first‐line ICI‐based systemic therapy (ICI plus chemotherapy [ICI + CT] or ICI + CT plus bevacizumab [ICI + CT + Bev]) from June 2019 to June 2024. The intracranial progression‐free survival (icPFS), progression‐free survival (PFS), and overall survival (OS) were compared between treatment groups and by BM local therapy. The PD‐L1 tumor proportion score (TPS) expression was evaluated for correlation with intracranial efficacy.

**Results:**

A total of 36 patients were enrolled in the study. The intracranial objective response rate (icORR) was 70.6% (ICI + CT) versus 78.6% (ICI + CT + Bev) (*p* = 0.689), with no significant differences in icPFS, PFS, or OS between the two different first‐line systemic regimens (all *p* > 0.05). Local BM therapy (*n* = 18) did not improve icPFS and OS (all *p* > 0.05). Extracranial PD‐L1 (TPS ≥ 50%, *n* = 13) correlated with superior icPFS, PFS, and OS (all *p* < 0.05) versus PD‐L1 TPS < 50%. Multivariate analysis confirmed PD‐L1 ≥ 50% as an independent prognostic factor (HR = 0.155; 95% CI, 0.025–0.939; *p* = 0.042).

**Conclusions:**

Adding bevacizumab to first‐line ICI‐chemotherapy did not enhance survival outcomes. Local treatment for BM did not provide additional survival advantages when combined with first‐line ICI‐based systemic therapy. Extracranial PD‐L1 TPS ≥ 50% predicted improved intracranial efficacy.

## Graphical abstract

The key question of this study is “what is the optimal first‐line therapy for driver gene‐negative NSCLC with brain metastases (BM), and can PD‐L1 predict intracranial efficacy?” This is a retrospective analysis of 36 patients treated with immune checkpoint inhibitor (ICI) + chemotherapy (CT) (*n* = 21) and ICI + CT + bevacizumab (Bev) (*n* = 15). This study evaluated icPFS, PFS, OS, and the impact of local BM therapy (*n* = 18). PD‐L1 TPS was assessed for correlation with outcomes. No significant difference was seen in icORR (70.6% vs. 78.6%, *p* = 0.689), icPFS, PFS, or OS between ICI + CT and ICI + CT + Bev. Local BM therapy did not improve survival (*p* > 0.05). Biomarkers used are PD‐L1 TPS ≥ 50% (*n* = 13) associated with longer icPFS, PFS, and OS (all *p* < 0.05) and independent prognostic factor (HR = 0.155, *p* = 0.042). Adding bevacizumab to ICI + CT did not improve survival. Local BM therapy offered no additional benefit with systemic ICI‐based treatment. PD‐L1 TPS ≥ 50% predicts superior intracranial efficacy.

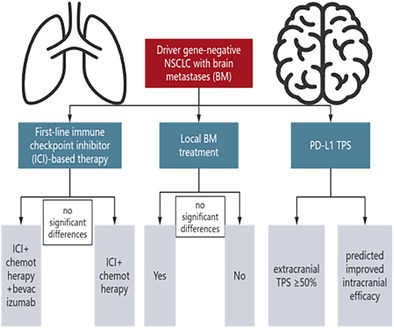

## 1 Introduction

About 30% of solid tumors will metastasize to the brain, and lung cancer accounts for half of them [1]. The prevalence of brain metastases (BM) in patients diagnosed with advanced non‐small cell lung cancer (NSCLC) was 28.4%, among which the prevalence of BM in *EGFR, ALK, ROS1* gene mutations, and wild‐type (excluding *EGFR, ALK, ROS1, KRAS*, and *RET* mutations) was 29.4%, 34.9%, 30.1%, and 28.8%, respectively [2]. BM is also a significant cause of death in NSCLC patients [2].

Regarding systemic treatment, due to the presence of the blood‐brain barrier, traditional chemotherapy is not ideal for treating intracranial lesions [3]. For patients with targetable targets, the current new targeted drugs have good intracranial efficacy [4, 5]. Using *EGFR* or *ALK* inhibitors with central nervous system penetration can delay the time for brain radiotherapy [4]. However, for NSCLC patients with BM who have driver gene‐negative or lack targetable therapeutic options, the current first‐line systemic treatment strategies include immunotherapy, especially immune checkpoint inhibitor (ICI), chemotherapy, and anti‐angiogenic therapy [6]. Notably, the efficacy of ICI in this population remains controversial. Some studies have indicated that ICI alone is effective [7], while others indicate that single‐agent ICI is insufficient, necessitating the combination of ICI with chemotherapy or dual immunotherapy [8]. The optimal treatment approach remains undetermined.

Surgery and brain radiotherapy are commonly used local treatments for BM, especially in patients with symptomatic BM [9, 10]. Due to the invasiveness of surgery and its strict indications, more patients receive brain radiotherapy. Stereotactic radiosurgery (SRS) offers superior disease control and reduces cognitive decline compared to whole‐brain radiotherapy (WBRT) [11]. However, it remains uncertain whether adding local BM treatment to first‐line ICI‐based systemic therapy confers additional intracranial control or survival benefits.

PD‐L1 expression is closely associated with the efficacy of ICI in NSCLC, with higher expression levels generally correlating with better outcomes [12]. However, due to the challenges in obtaining BM samples for PD‐L1 testing, the potential of extracranial PD‐L1 expression as a predictor of intracranial immunotherapy efficacy warrants further investigation.

Clinical trials usually exclude patients with performance status (PS) scores > 2 or those with symptomatic BM [13]. Consequently, the treatment strategies for this subgroup can only be explored through retrospective real‐world studies. This study aims to preliminarily assess the efficacy of first‐line immune‐based combination therapy in patients with BM from driver gene‐negative NSCLC. The analysis includes examining the differences between various first‐line immune combination regimens, evaluating whether the addition of local BM treatment offers further benefits, and exploring the relationship between extracranial PD‐L1 expression and intracranial therapeutic efficacy.

## 2 Materials and Methods

### 2.1 Study Patients

This study enrolled patients with advanced NSCLC admitted to the Cancer Hospital of the Chinese Academy of Medical Sciences from June 2019 to June 2024. Inclusion criteria were: (a) new diagnosis with NSCLC confirmed by histopathology; (b) contrast‐enhanced cranial magnetic resonance imaging (MRI) diagnosed brain metastases at the time of initial treatment; (c) non‐squamous carcinoma received a driven gene test, which included *EGFR, ALK*, and *ROS1*; (d) receiving at least one injection of first‐line systemic therapy based on ICI; and (e) measurable lesions according to the Response Evaluation Criteria in Solid Tumors version 1.1 (RECIST v1.1). Exclusion criteria were: (a) patients with targetable therapy‐driven gene alterations, including *EGFR, ALK*, and *ROS1*; (b) second primary malignant tumor within 20 years; (c) received an organ transplant or used enhanced immunosuppressants; and (d) history of psychiatric illness. The clinical pathological characteristics of patients were obtained through the electronic medical record system. The researchers carefully evaluated the efficacy according to the evaluation criteria and collected survival information through detailed telephone follow‐ups. The data cutoff date of this study was March 2, 2025. In the event of loss to follow‐up, the most recent follow‐up date was used as the data cutoff. This study design and analysis procedures were performed in accordance with the principles outlined in the Declaration of Helsinki (as revised in 2013) and approved by the Ethics Committee of the National Cancer Center/National Clinical Research Center for Cancer/Cancer Hospital, Chinese Academy of Medical Sciences and Peking Union Medical College (No. 2025022418250802).

### 2.2 Efficacy Evaluation

The systemic therapy efficacy was evaluated by the RECIST v1.1, while the therapeutic response of intracranial lesions was assessed using the modified RECIST (mRECIST) criteria [14]. According to the evaluation criteria, the response to lesions was categorized into four types, including complete response (CR), partial response (PR), stable disease (SD), and progressive disease (PD). mRECIST acquired that the maximum diameter of intracranial lesions should be more than or equivalent to 5 mm and incorporate up to five lesions. Intracranial objective response rate (icORR) represents the proportion of intracranial lesions with CR and PR. The intracranial disease control rate (icDCR) represents the proportion of intracranial lesions with CR, PR, and SD.

### 2.3 Study Endpoints

The study endpoints encompassed evaluating the efficacy of different first‐line systemic therapies, whether local BM treatment was administered, and the impact of varying levels of PD‐L1 expression on the efficacy of treatment for both intracranial and extracranial lesions. Progression‐free survival (PFS) was defined as the time from initiating systemic treatment to disease progression, death, or the end of follow‐up. Intracranial PFS (icPFS) was defined as the time from the start of treatment, including cerebral local therapy and systemic therapy, to the progression of intracranial lesions, death from any cause, or the end of follow‐up. Overall survival (OS) was defined as the time from histopathology diagnosis to death from any cause or the end of follow‐up.

### 2.4 Statistical Analyses

Descriptive statistics were used to show the distribution of patients and baseline demographic and clinical characteristics. The Kaplan–Meier method and the corresponding survival curves were used to analyze icPFS, PFS, and OS. The median follow‐up time was calculated using the reverse Kaplan–Meier method. Fisher's exact test was utilized for categorical variables. The least absolute shrinkage and selection operator (LASSO) regression was employed to select significant variables influencing prognosis, and the regularization path plot was utilized to demonstrate the relative importance of these variables visually. A cross‐validation plot was applied to determine the optimal regularization parameter. Cox proportional hazards regression was adopted to identify key variables across different subgroups. Variables with a *p* value < 0.1 in the univariate Cox regression analysis were included in the multivariate Cox regression model. All statistical analyses were performed using RStudio software (V.4.3.1; R Core Team, Vienna, Austria). All statistical tests were two‐tailed, and a *p* value < 0.05 indicated statistical significance.

## 3 Results

### 3.1 Patient Characteristics at Baseline

Between June 2019 and June 2024, 36 patients who met the eligible criteria were enrolled in this study. The baseline patients' characteristics are shown in Table 1. Among them, 13 (36.1%) patients were diagnosed with NSCLC at the age of ≥ 65 years, and the majority were male (91.7%). Of the 36 patients, 10 (27.8%) had a PS score ≥ 2. Most patients had a history of smoking, including those who had quit (61.1%) and those who had not quit (22.2%). Adenocarcinoma accounted for most pathological types (63.9%), followed by squamous cell carcinoma (27.8%), and three were other pathological types, including two adenosquamous carcinomas and one large cell neuroendocrine carcinoma. *KRAS* gene mutations were detected in nine patients (25.0%), *TP53* mutations were detected in 14 patients (38.9%), and the rest were not checked or not detected. Among the 36 cases, 21 patients underwent PD‐L1 tumor proportion score (TPS) testing, of which approximately 2/3 of the patients had an expression of > 50%; 11 patients underwent tumor mutational burden (TMB) testing, and about half of the patients had a TMB of ≥ 10 Mut/Mb. In addition to BM, patients also had metastases in other parts of the body, including 19.4% of patients with liver metastases, 1/3 of patients with lung metastases, 22.2% with adrenal metastases, and 19.4% with bone metastases. About half (47.2%) of the patients had symptomatic BM, and most had multiple BM (52.8%).

**TABLE 1:** Demographic and clinical characteristics of patients at baseline.

Characteristics	Values (*N* = 36)
Age, *n* (%)	
≥ 65 years	13 (36.1)
< 65 years	23 (63.9)
Gender, *n* (%)	
Female	3 (8.3)
Male	33 (91.7)
ECOG performance status, *n* (%)	
< 2	26 (72.2)
≥ 2	10 (27.8)
Smoking history, *n* (%)	
Current	8 (22.2)
Former	22 (61.1)
Never	6 (16.7)
Histopathology, *n* (%)	
Adenocarcinoma	23 (63.9)
Squamous carcinoma	10 (27.8)
Other	3 (8.3)
*KRAS* mutation status, *n* (%)	
No/Unknown	27 (75.0)
Yes	9 (25.0)
*TP53* mutation status, *n* (%)	
No/Unknown	22 (61.1)
Yes	14 (38.9)
PD‐L1 TPS, *n* (%)	
< 50%	8 (22.2)
≥ 50%	13 (36.1)
Unknown	15 (41.7)
TMB, mutations per megabase, *n* (%)	
< 10	5 (13.9)
≥ 10	6 (16.7)
Unknown	25 (69.4)
Liver metastases, *n* (%)	
No	29 (80.6)
Yes	7 (19.4)
Lung metastases, *n* (%)	
No	24 (66.7)
Yes	12 (33.3)
Adrenal metastases, *n* (%)	
No	28 (77.8)
Yes	8 (22.2)
Bone metastases, *n* (%)	
No	29 (80.6)
Yes	7 (19.4)
Number of BM, *n* (%)	
Multiple	19 (52.8)
Single	17 (47.2)
Neurological symptoms, *n* (%)	
No	19 (52.8)
Yes	17 (47.2)
First‐line treatment, *n* (%)	
ICI + CT	21 (58.3)
ICI + CT + Bev	15 (41.7)
Thoracic radiotherapy, *n* (%)	
No	31 (86.1)
Yes	5 (13.9)
Local BM treatment, *n* (%)	
No	18 (50.0)
Yes	18 (50.0)

Abbreviations: Bev, bevacizumab; BM, brain metastases; CT, chemotherapy; ECOG, Eastern Cooperative Oncology Group; ICI, immune checkpoint inhibitor; PD‐L1 TPS, programmed death‐ligand 1 tumor proportion score; TMB, tumor mutation burden.

Regarding treatment, approximately half of the patients received local treatment for BM, including two patients who underwent surgical intervention for BM and 16 patients who received brain radiotherapy. The first‐line systemic therapy was treated with ICI‐based combination therapies, of which 21 patients (53.8%) received combined ICI and chemotherapy (ICI + CT); 15 received combined ICI plus chemotherapy and bevacizumab (ICI + CT + Bev). The ICI in this study was all programmed cell death protein 1 (PD‐1) / programmed death‐ligand 1 (PD‐L1) monoclonal antibody treatment, including 8 PD‐L1 monoclonal antibodies and 28 cases of PD‐1 monoclonal antibodies. Five patients discontinued the drug due to immune‐related adverse reactions, including three cases of grade 2 immune‐related pneumonia, one case of grade 3 adrenal cortical insufficiency, and one case of immune‐related nephritis.

### 3.2 Intracranial Lesions Response Rate

According to mRECIST criteria, 31 patients were eligible for assessment of the intracranial treatment response. Among them, the ICI + CT treatment group had an icORR of 70.59% (12 of 17) and icDCR of 94.12% (16 of 17). In the ICI + CT + Bev group, the icORR was 78.57% (11 of 14), and the icDCR was 100%. The difference in icORR between the two groups was not statistically significant (*p* = 0.689), nor was the difference in icDCR (*p* = 1.000).

The icORR of patients receiving local treatment for BM was 58.82% (10 of 17), with an icDCR of 94.12% (16 of 17), whereas those who did not receive local treatment had an icORR and icDCR of 92.86% and 100%, respectively. The differences in icDCR between the two groups were not statistically significant (*p* = 1.000). Notably, the icORR between the two groups was a statistically significant difference (*p* = 0.045).

### 3.3 Survival Analysis

**FIGURE 1:**
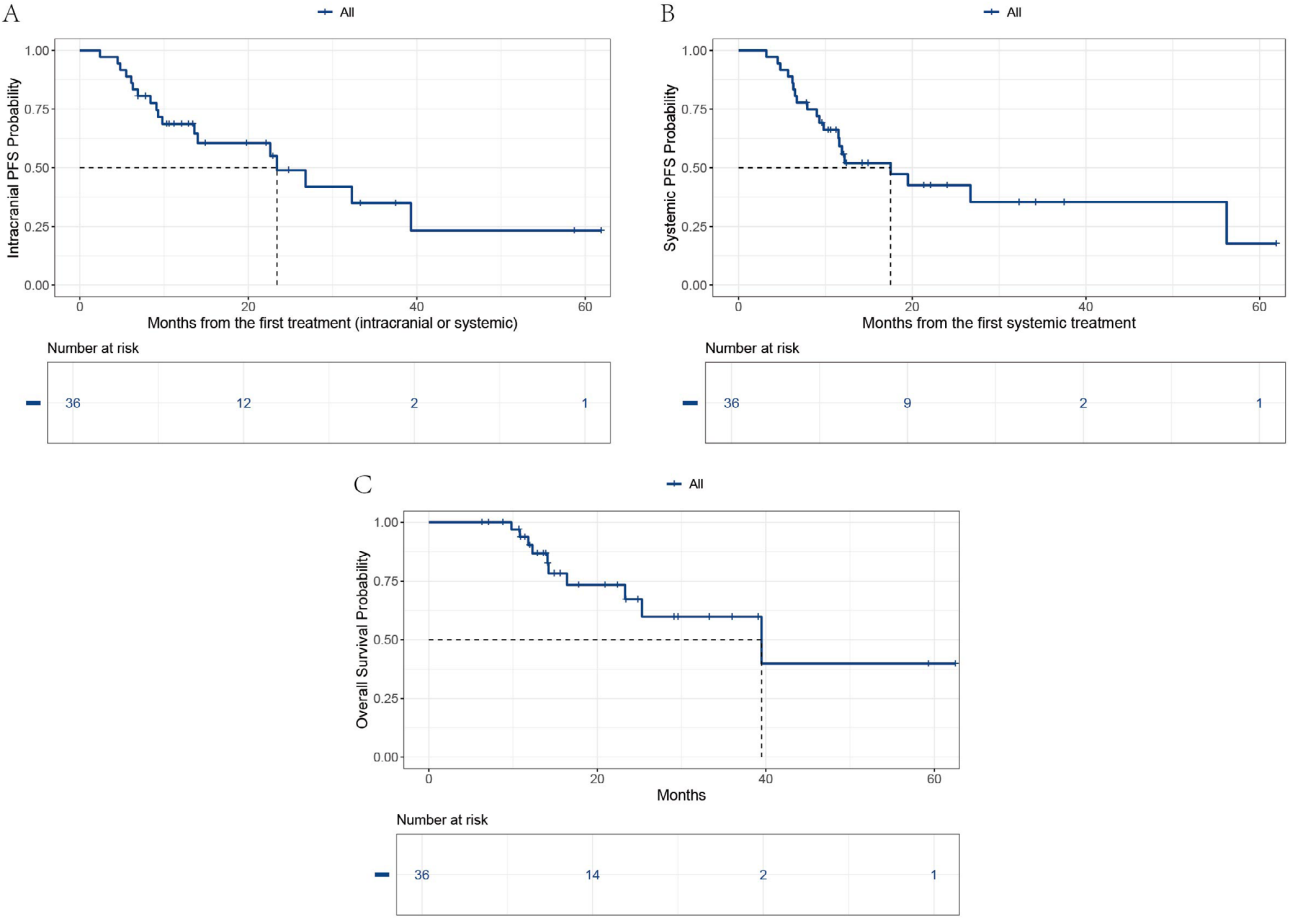
Kaplan–Meier plots for icPFS (A), PFS (B), and OS (C) in the overall population. icPFS, intracranial progression‐free survival; OS, overall survival; PFS, progression‐free survival.

**FIGURE 2:**
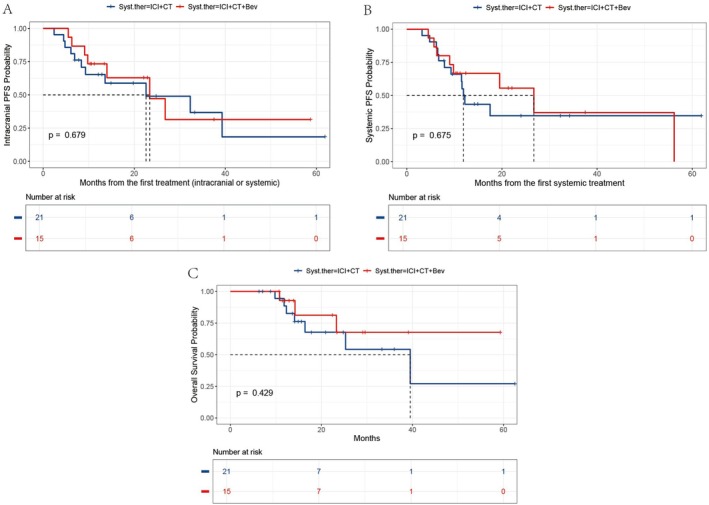
Kaplan–Meier analysis revealed no significant differences in icPFS (A), PFS (B), or OS (C) between the ICI + CT and ICI + CT + Bev groups (all log‐rank *p* > 0.05). Bev, bevacizumab; CT, chemotherapy; ICI, immune checkpoint inhibitor; icPFS, intracranial progression‐free survival; OS, overall survival; PFS, progression‐free survival; Syst.ther, systemic therapy.

The median follow‐up time in this study was 22.4 months (95% CI: 14.9–39.1). In the overall population, the median icPFS, PFS, and OS were 23.4 months (95% CI: 14.0, not reached), 17.5 months (95% CI: 11.5, not reached), and 39.5 months (95% CI: 25.3, not reached), respectively (Figure 1). We explored the impact of different first‐line immune combination therapy regimens on the survival of patients with driver gene‐negative NSCLC with BM. The Kaplan–Meier survival curves revealed that the *p* values from the log‐rank tests for icPFS, PFS, and OS between the ICI + CT and ICI + CT + Bev groups were 0.679, 0.675, and 0.429, respectively, indicating no statistically significant differences (Figure 2).

**FIGURE 3:**
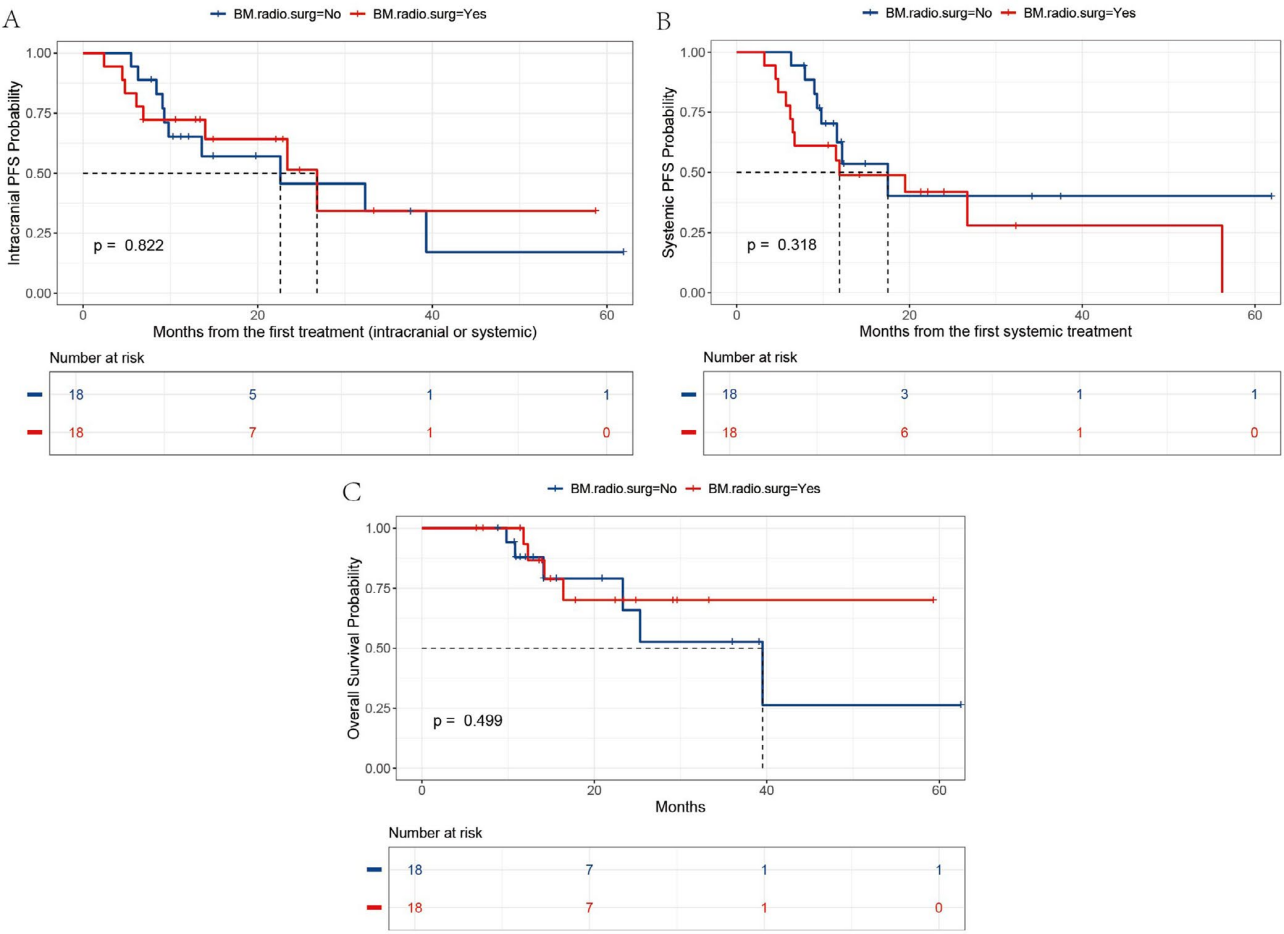
Kaplan–Meier analysis revealed no significant differences in icPFS (A), PFS (B), or OS (C) between patients who received local BM treatment (surgery or radiotherapy) and those who did not (all log‐rank *p* > 0.05). BM.radio.surg, radiotherapy or surgery for brain metastases; icPFS, intracranial progression‐free survival; OS, overall survival; PFS, progression‐free survival.

We further investigated the impact of local treatment on survival in the context of combined immunotherapy. The log‐rank test *p* values for icPFS, PFS, and OS, based on whether local BM treatment was administered, were 0.822, 0.318, and 0.499, respectively, all of which were not statistically significant (Figure 3).

**FIGURE 4:**
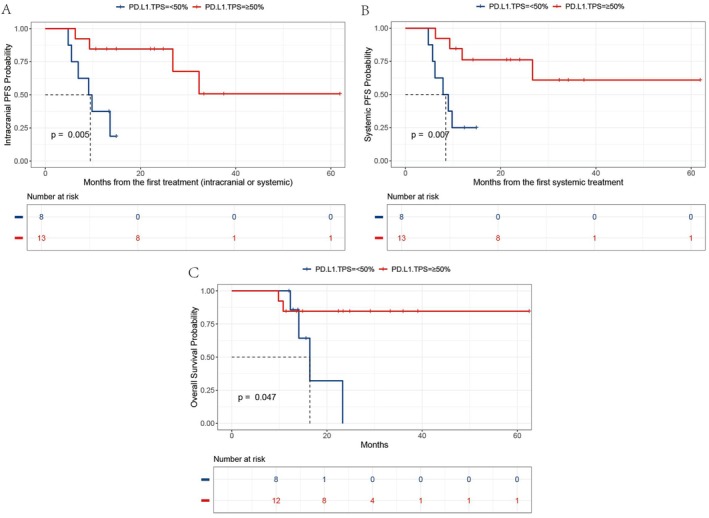
Kaplan–Meier analysis revealed that the PD‐L1 high‐expression group (TPS ≥ 50%) had better icPFS (A), PFS (B), and OS (C) outcomes than the low‐expression group (TPS < 50%). icPFS, intracranial progression‐free survival; PD.L1.TPS, programmed death‐ligand 1 tumor proportion score; OS, overall survival; PFS, progression‐free survival.

Whether PD‐L1 expression levels in extracranial lesions can predict therapeutic response for intracerebral metastases remains unclear. We aim to address this gap by evaluating PD‐L1 expression in extracranial tissue from 21 patients. Among these patients, 13 demonstrated PD‐L1 TPS ≥ 50%, while eight showed TPS < 50%. Survival result analysis showed that there were significant differences in icPFS, PFS, and OS between the PD‐L1 TPS ≥ 50% group and the TPS < 50% group, with *p* values of 0.005, 0.007, and 0.047, respectively (Figure 4). The results indicate that patients with high PD‐L1 expression (TPS ≥ 50%) experienced superior intracranial and extracranial benefits compared to those with lower expression levels (TPS < 50%).

### 3.4 Prognostic Variables

**FIGURE 5:**
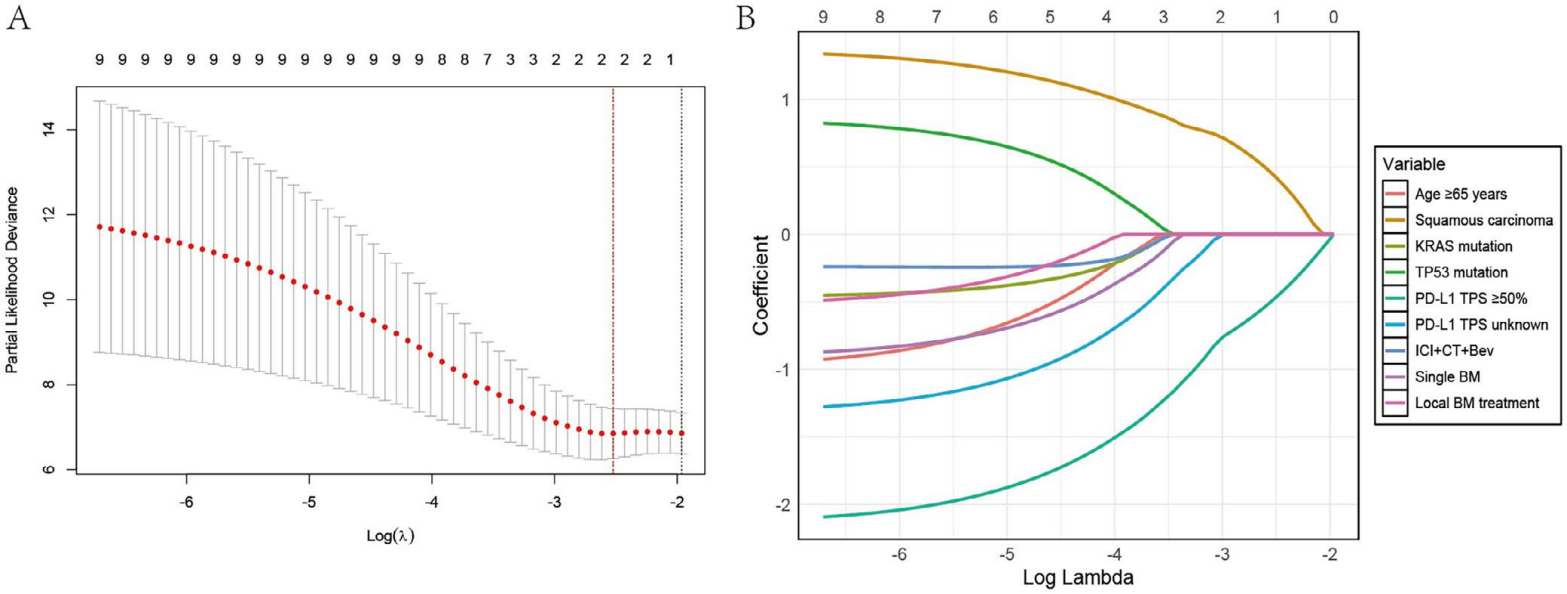
The LASSO regression analysis was employed to identify significant prognostic variables. The best regularization parameter (*λ* value) in the cross‐validation graph (A). The regularization path graph (B) showed that PD‐L1 TPS ≥ 50% was the most crucial variable. Bev, bevacizumab; BM, brain metastases; CT, chemotherapy; ICI, immune checkpoint inhibitor; PD‐L1 TPS, programmed death‐ligand 1 tumor proportion score.

In the prognostic analysis, we incorporated eight key variables: age, histological type, *KRAS* mutation status, *TP53* mutation status, PD‐L1 expression, first‐line immune‐combination regimen, whether intracranial lesions were multiple, and whether BM received local treatment. LASSO regression analysis identified the optimal regularization parameter based on the cross‐validation plot (Figure 5A). The regularization path plot (Figure 5B) revealed that PD‐L1 TPS ≥ 50% was the most influential variable. Multivariate Cox analysis indicated that patients with extracranial PD‐L1 TPS ≥ 50% exhibited a significantly reduced mortality risk compared to those with PD‐L1 TPS < 50% with a *p* value of 0.042 (HR = 0.155, 95% CI: 0.025–0.939) (Table 2). The Cox regression analyses yielded consistent results for both PFS (Table 3) and icPFS (Table 4).

**TABLE 2:** Univariate and multivariate analysis of overall survival for patients with BM from driver gene‐negative NSCLC (*N* = 36).

Characteristics	Univariate analysis			Multivariate analysis		
	HR	95% CI	*p*	HR	95% CI	*p*
Age, years						
≥ 65	Reference					
< 65	0.739	0.180–3.040	0.675			
Histopathology						
Squamous carcinoma	Reference					
Other	0.364	0.099–1.341	0.129			
*KRAS* mutation status						
No/Unknown	Reference					
Yes	0.342	0.043–2.749	0.313			
*TP53* mutation status						
No/Unknown	Reference					
Yes	1.307	0.321–5.330	0.709			
PD‐L1 TPS						
< 50%	Reference			Reference		
≥ 50%	0.155	0.025–0.939	0.042*	0.155	0.025–0.939	0.042*
Unknown	0.345	0.073–1.626	0.178	0.345	0.073–1.626	0.178
First‐line treatment						
ICI + CT	Reference					
ICI + CT + Bev	0.582	0.150–2.261	0.434			
Number of BM						
Single	Reference					
Multiple	0.911	0.258–3.214	0.885			
Local BM treatment						
No/Unknown	Reference					
Yes	0.646	0.180–2.314	0.502			

*Note:* Only variable PD‐L1 TPS was included in the multivariate analysis; therefore, the results of univariate and multivariate analyses are identical.

Abbreviations: Bev, bevacizumab; BM, brain metastases; CT, chemotherapy; ICI, immune checkpoint inhibitor; NSCLC, non‐small cell lung cancer; PD‐L1 TPS, programmed death‐ligand 1 tumor proportion score.

* *p* < 0.05.

**TABLE 3:** Univariate and multivariate analysis of progression‐free survival for patients with BM from driver gene‐negative NSCLC (*N* = 36).

Characteristics	Univariate analysis			Multivariate analysis		
	HR	95% CI	*p*	HR	95% CI	*p*
Age, years						
≥ 65	Reference					
< 65	0.566	0.219–1.461	0.239			
Histopathology						
Squamous carcinoma	Reference					
Other	0.735	0.276–1.960	0.539			
*KRAS* mutation status						
No/Unknown	Reference					
Yes	0.416	0.121–1.430	0.164			
*TP53* mutation status						
No/Unknown	Reference					
Yes	1.624	0.643–4.099	0.305			
PD‐L1 TPS						
< 50%	Reference			Reference		
≥ 50%	0.136	0.034–0.537	0.004*	0.136	0.034–0.537	0.004*
Unknown	0.546	0.190–1.569	0.261	0.546	0.190–1.569	0.261
First‐line treatment						
ICI + CT	Reference					
ICI + CT + Bev	0.825	0.335–2.030	0.676			
Number of BM						
Single	Reference					
Multiple	0.913	0.377–2.212	0.840			
Local BM treatment						
No/Unknown	Reference					
Yes	1.579	0.640–3.894	0.322			

*Note:* Only variable PD‐L1 TPS was included in the multivariate analysis; therefore, the results of univariate and multivariate analyses are identical.

Abbreviations: Bev, bevacizumab; BM, brain metastases; CT, chemotherapy; ICI, immune checkpoint inhibitor; NSCLC, non‐small cell lung cancer; PD‐L1 TPS, programmed death‐ligand 1 tumor proportion score.

* *p* < 0.05.

**TABLE 4:** Univariate and multivariate analysis of intracranial progression‐free survival for patients with BM from driver gene‐negative NSCLC (*N* = 36).

Characteristics	Univariate analysis			Multivariate analysis		
	HR	95% CI	*p*	HR	95% CI	*p*
Age, years						
≥ 65	Reference					
< 65	0.575	0.211–1.567	0.279			
Histopathology						
Squamous carcinoma	Reference					
Other	0.781	0.274–2.224	0.643			
*KRAS* mutation status						
No/Unknown	Reference					
Yes	0.488	0.140–1.706	0.261			
*TP53* mutation status						
No/Unknown	Reference					
Yes	1.407	0.523–3.785	0.499			
PD‐L1 TPS						
< 50%	Reference			Reference		
≥ 50%	0.140	0.034–0.568	0.006*	0.140	0.034–0.568	0.006*
Unknown	0.377	0.118–1.205	0.100	0.377	0.118–1.205	0.100
First‐line treatment						
ICI + CT	Reference					
ICI + CT + Bev	0.818	0.316–2.118	0.679			
Number of BM						
Single	Reference					
Multiple	0.960	0.376–2.454	0.932			
Local BM treatment						
No/Unknown	Reference					
Yes	0.898	0.352–2.292	0.822			

*Note:* Only variable PD‐L1 TPS was included in the multivariate analysis; therefore, the results of univariate and multivariate analyses are identical.

Abbreviations: Bev, bevacizumab; BM, brain metastases; CT, chemotherapy; ICI, immune checkpoint inhibitor; NSCLC, non‐small cell lung cancer; PD‐L1 TPS, programmed death‐ligand 1 tumor proportion score.

* *p* < 0.05.

## 4 Discussion

Our study showed that for NSCLC patients with driver gene‐negative BM, adding bevacizumab to first‐line ICI‐chemotherapy combined regimens did not improve survival outcomes. Many studies have confirmed that ICI should be considered for patients with BM from NSCLC with driver gene‐negative [15, 16, 17]. A meta‐analysis showed that adding chemotherapy significantly improved OS when combined with ICI (HR = 0.58) or dual ICI (PD‐1/PD‐L1 inhibitor plus cytotoxic T‐lymphocyte‐associated protein 4 [CTLA‐4] inhibitor) (HR = 0.61) versus corresponding monotherapies [8]. Although ICI‐chemotherapy combinations have established efficacy in the first‐line treatment of driver mutation‐negative NSCLC with BM, the potential synergistic benefit of incorporating anti‐angiogenic agents into this regimen remains investigational. A meta‐analysis suggested that vascular endothelial growth factor (VEGF) also plays a role in preventing brain metastases in patients [18]. The IMpower150 study [19] suggested that the four‐drug combination of ICI plus bevacizumab and chemotherapy might delay intracranial lesion progression compared to bevacizumab‐chemotherapy alone. However, the difference did not reach statistical significance. The high proportion of PD‐L1‐high (TPS ≥ 50%) patients in our cohort may explain the lack of observed benefit from bevacizumab, as the pronounced efficacy of the immunotherapy‐chemotherapy combination likely obscured any potential incremental benefit from VEGF inhibition. However, further large‐sample prospective studies are needed to confirm this view.

In this study, the response rate of intracranial lesions to first‐line systemic treatment in both groups was high, with icORR exceeding 70% and icDCR exceeding 90%, which is similar to previous studies [20], considering that all patients included in this study were first‐line ICI‐based therapy patients, rather than second‐line or later patients.

In patients with driver gene‐negative accompanied by BM in NSCLC, the addition of local therapy (surgery or radiotherapy) to first‐line ICI‐based combination therapy failed to demonstrate significant survival benefits. The observation may be related to the fact that there was no pure ICI group in this study, as all were ICI combination groups (combination with chemotherapy with or without bevacizumab). Former studies have shown that radiotherapy can improve survival for the ICI single‐agent group, but this benefit was not seen in the ICI combination chemotherapy group [21, 22]. Our study uncovered a particularly intriguing finding: the icORR and icDCR were numerically lower in the radiotherapy group than in the non‐radiotherapy group. Notably, this difference reached statistical significance. It may be related to the substantial reduction in the proportion of immune cells in BM after brain radiotherapy, including the depletion of tissue‐resident macrophages and the infiltration of pro‐inflammatory monocytes, thus affecting the efficacy of ICI [23].

Additionally, PD‐L1 expression is a well‐established predictor of ICI efficacy in NSCLC [12]. Moutafi et al. found that PD‐L1 expression was lower in BM than in primary lung lesions [24]. However, obtaining intracranial tissue for testing remains clinically challenging. Our study showed that PD‐L1 TPS expression in extracranial lesions can reflect the efficacy of intracranial therapy, with patients having PD‐L1 TPS ≥ 50% showing better icPFS and OS. This finding was further validated by LASSO regression and Cox proportional hazards regression analyses (Figure 5). Previous studies have found that PD‐L1 expression was associated with better OS but not with icPFS when ICI was used after local brain treatment (radiotherapy or surgery) and before the progression of intracranial lesions [25]. This finding may suggest that local treatment for BM did not provide icPFS benefits in patients with BM with high PD‐L1 expression, but further prospective studies were needed to confirm it.

Many clinical trials systematically exclude patients with poor performance status (PS) or symptomatic BM, leading to a significant gap in the data concerning this population [26, 27]. Real‐world studies are essential to bridge this gap. In our study, 10 (27.8%) patients had a PS score of ≥ 2, and 17 (47.2%) patients presented with symptoms related to BM. Of 19 patients with progression after first‐line therapy evaluated by RECIST v1.1, only two had isolated intracranial progression, three had combined intra/extracranial progression, while most (14/19) showed purely extracranial progression. However, extended follow‐up was necessary to delineate more nuanced treatment patterns and to optimize therapeutic approaches in this population.

This study has some limitations. As a retrospective study, bias is inevitable. Additionally, the small sample size and insufficient follow‐up time mean that the survival outcomes should be interpreted cautiously. Although MRI confirmed baseline brain metastases in all cases, the use of computed tomography for subsequent response assessment in some patients represents a study limitation. Prospective trials should implement uniform MRI‐based evaluation protocols. The optimal first‐line systemic treatment approach for NSCLC patients with driver gene‐negative BM requires further investigation. Future studies should incorporate expanded subgroup analyses to identify optimal treatment strategies for distinct patient populations, including monotherapy, dual immune checkpoint inhibition, combination regimens with anti‐angiogenic agents, chemotherapy, and antibody‐drug conjugates (ADCs). Although this study suggests that combining local treatment for BM with immunotherapy‐based systemic therapy did not demonstrate significant clinical benefit in this patient cohort, the differential effects of various radiotherapy modalities and optimal sequencing between systemic treatment and radiotherapy remain to be elucidated. Furthermore, the complex interactions between surgical resection, radiotherapy techniques, and systemic therapies in managing BM require more extensive investigation.

## 5 Conclusion

In this study, we preliminarily analyzed first‐line ICI‐based combination therapies in driver gene‐negative NSCLC patients with BM. Our study demonstrated that adding bevacizumab to first‐line ICI + CT failed to provide additional intracranial benefits or OS improvement in this patient population. Notably, adding local BM therapy to systemic treatment failed to confer additional clinical benefit. Furthermore, PD‐L1 expression in extracranial lesions showed potential as a surrogate marker for intracranial treatment response. These findings inform therapeutic decision‐making for this challenging patient population.

## Author Contributions

Conception and design: Mengxing You, Puyuan Xing, and Junling Li. Collection and assembly of data: Lige Wu, Jiayu Liu, Hanqi Yuan, and Zihe Wang. Provision of study materials or patients: Xuezhi Hao, Puyuan Xing, and Junling Li. Data analysis and interpretation: Mengxing You. Manuscript writing: All authors. Final approval of manuscript: All authors.

## Conflicts of Interest

The authors declare no conflicts of interest.

## Data Availability

The data sets generated during and/or analyzed during this study are available from the corresponding author upon reasonable request.
